# Supplementary material for: Genome sequence-based identification of Enterobacter strains and description of Enterobacter pasteurii sp. nov
Source: Microbiol Spectr. 2023 Dec 15;12(1):e03150-23. doi: 10.1128/spectrum.03150-23 (PMC10783019; doi:10.1128/spectrum.03150-23)
Supplement: Supplemental figures — Fig. S1 to S4. [file spectrum.03150-23-s0001.pdf]

Supplementary Data

Genome Sequence-Based Identification of *Enterobacter* Strains and Description of *Enterobacter pasteurii* sp. nov.

Praveen Rahi[1], Estelle Muhle[1], Cyril Scandola[2], Gerald Touak[1], Dominique Clermont[1]

[1]Institut Pasteur, Université Paris Cité, Collection of Institut Pasteur (CIP), F-75015 Paris, France

[2]Institut Pasteur, Université Paris Cité, Ultrastructural Bioimaging Unit, 75015 Paris, France

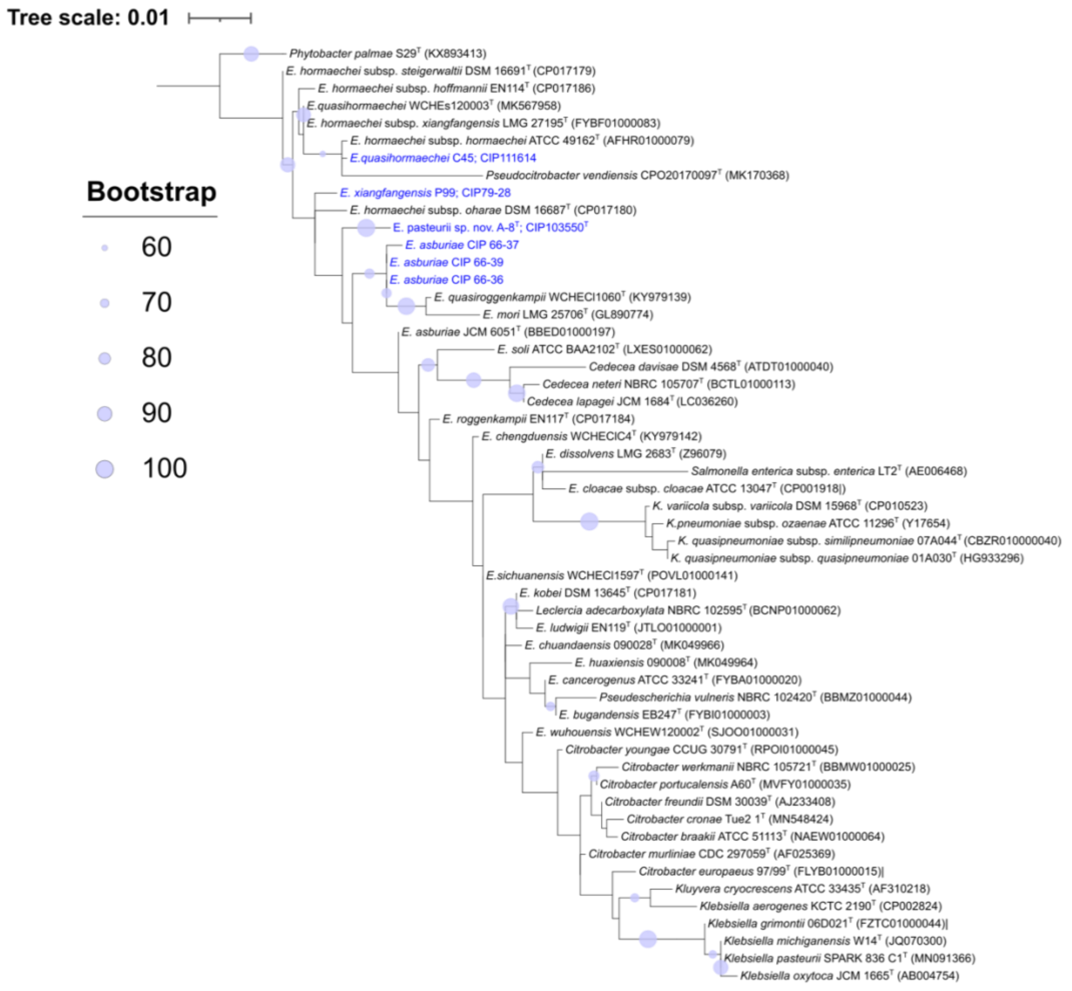

Fig S1. 16S rRNA gene phylogenetic tree obtained in IQ-TREE from the 16S rRNA gene sequences of six *Enterobacter* cloacae complex strains and their related taxa using maximum likelihood method. The circle on the nodes indicates the bootstrap value.

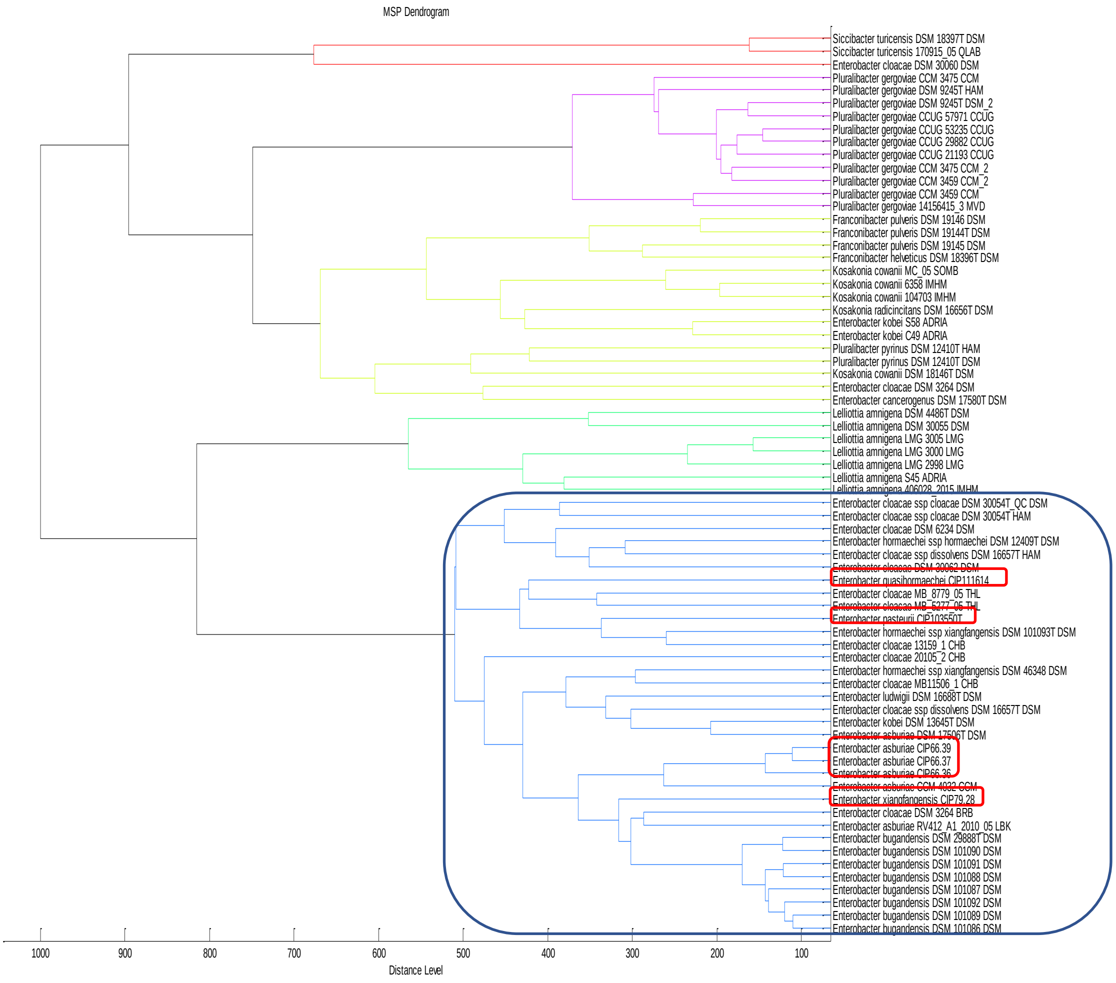

Fig S2: Dendrogram constructed based on MALDI-TOF MS profiles of *Enterobacter* strains and other members of *Enterobacteriaceae* family present in Bruker biotyper database.

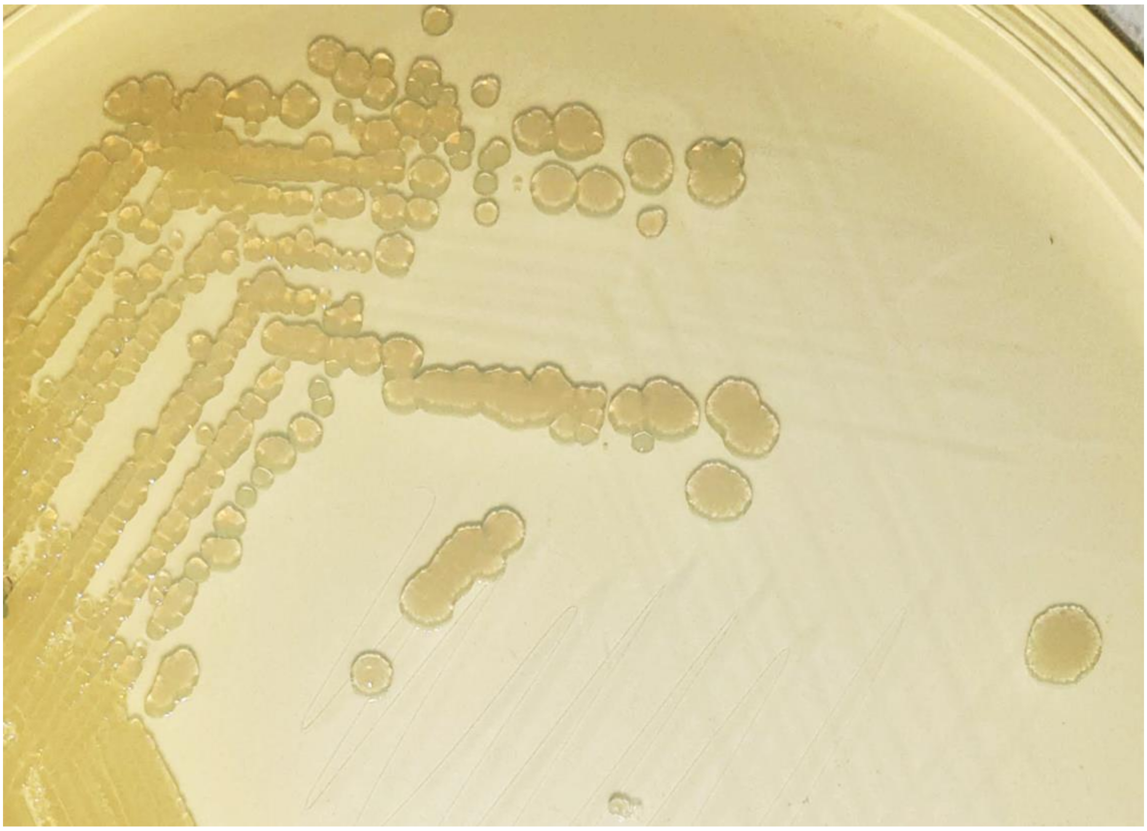

Fig S3. Colonies of strain A-8^T on tyripticase soy agar after the incubation of 24h at 37 °C.

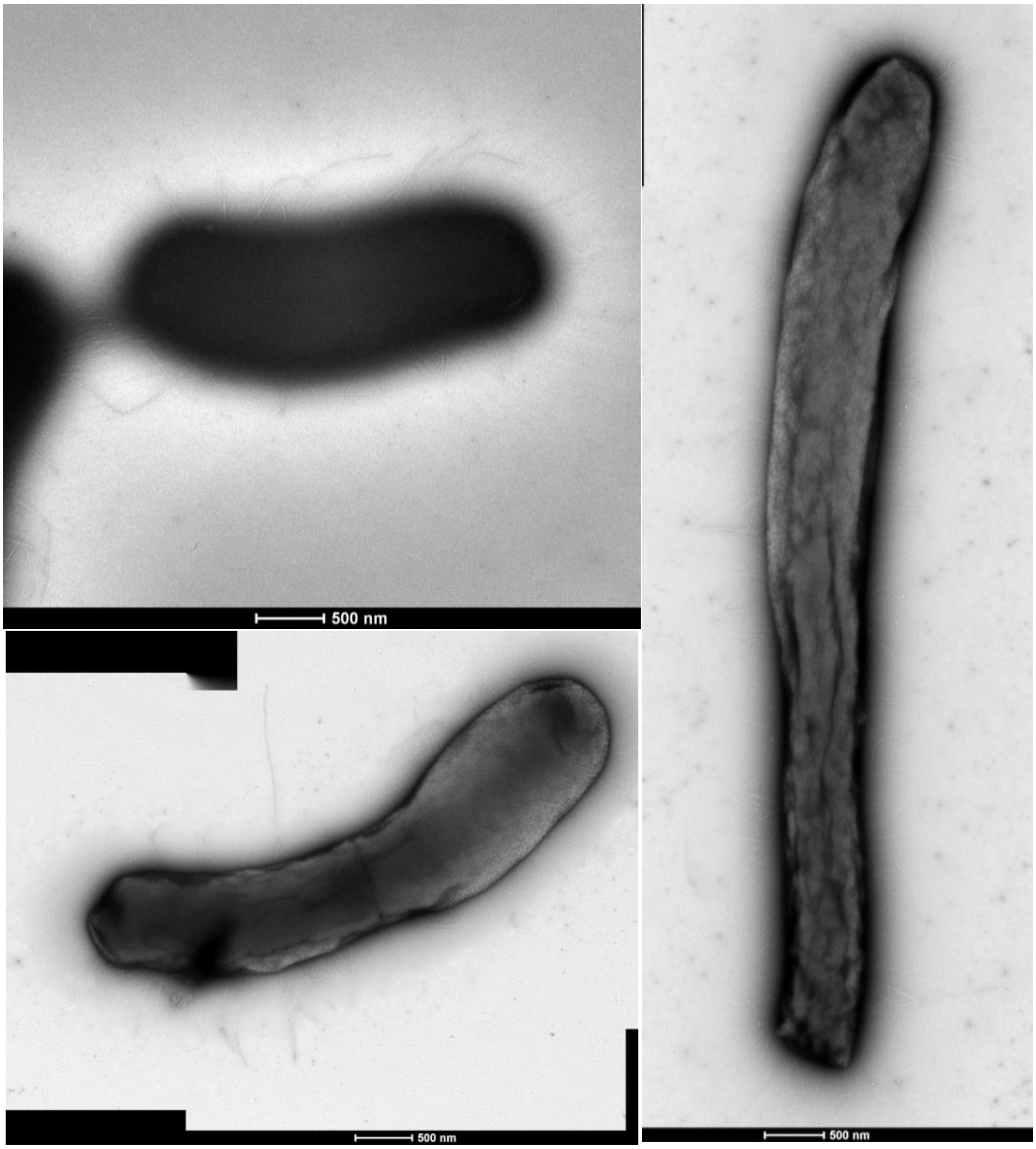

Fig S4: Transmission electron microscopy image of strain A-8^T after negative staining. Scale bar 500 nm.
